# Trastuzumab Decreases the Expression of G1/S Regulators and Syndecan-4 Proteoglycan in Human Rhabdomyosarcoma

**DOI:** 10.3390/ijms26052137

**Published:** 2025-02-27

**Authors:** Dora Julianna Szabo, Eniko Toth, Kitti Szabo, Zsofia Kata Hegedus, Noemi Bozsity-Farago, Istvan Zupko, Laszlo Rovo, Xue Xiao, Lin Xu, Aniko Keller-Pinter

**Affiliations:** 1 Department of Biochemistry, Faculty of Medicine, University of Szeged, 6720 Szeged, Hungary; 2 Centre of Excellence for Interdisciplinary Research, Development and Innovation, University of Szeged, 6720 Szeged, Hungary; 3 Institute of Pharmacodynamics and Biopharmacy, Faculty of Pharmacy, University of Szeged, 6720 Szeged, Hungary; 4 Department of Oto-Rhino-Laryngology and Head-Neck Surgery, University of Szeged, 6720 Szeged, Hungary; 5 Quantitative Biomedical Research Center, Department of Population and Data Sciences, University of Texas Southwestern Medical Center, Dallas, TX 75390, USA

**Keywords:** rhabdomyosarcoma, proteoglycan, syndecan-4, trastuzumab, cell cycle, MyoD

## Abstract

Rhabdomyosarcoma (RMS), the most common soft tissue sarcoma in children, arises from skeletal muscle cells that fail to differentiate terminally. Two subgroups of RMS, fusion-positive and fusion-negative RMS (FPRMS and FNRMS, respectively), are characterized by the presence or absence of the *PAX3/7-FOXO1* fusion gene. RMSs frequently exhibit increased expression of human epidermal growth factor receptor-2 (HER2). Trastuzumab is a humanized monoclonal antibody targeting HER2, and its potential role in RMS treatment remains to be elucidated. Syndecan-4 (SDC4) is a heparan sulfate proteoglycan (HSPG) affecting myogenesis via Rac1-mediated actin remodeling. Previously, we demonstrated that the SDC4 gene is amplified in 28% of human FNRMS samples, associated with high mRNA expression, suggesting a tumor driver role. In this study, after analyzing the copy numbers and mRNA expressions of other HSPGs in human RMS samples, we found that in addition to SDC4, syndecan-1, syndecan-2, and glypican-1 were also amplified and highly expressed in FNRMS. In RD (human FNRMS) cells, elevated SDC4 expression was accompanied by low levels of phospho-Ser179 of SDC4, leading to high Rac1-GTP activity. Notably, this high SDC4 expression in RD cells decreased following trastuzumab treatment. Trastuzumab decreased the levels of G1/S checkpoint regulators cyclin E and cyclin D1 and reduced the cell number; however, it also downregulated the cyclin-dependent kinase inhibitor p21. The level of MyoD, a transcription factor essential for RMS cell survival, also decreased following trastuzumab administration. Our findings contribute to the understanding of the role of SDC4 in FNRMS. Since HER2 is expressed in about half of RMSs, the trastuzumab-mediated changes observed here may have therapeutic implications.

## 1. Introduction

Rhabdomyosarcoma (RMS) is the most common soft tissue sarcoma in childhood and adolescence, with an incidence rate of 4.58/million under the age of 20 [1]. The 3-year survival rate of the metastatic form is still less than 30% [2]. RMS is characterized by impaired muscle differentiation, originating from skeletal myoblastic cells. The most common sites of RMS are the head and neck region, the genitourinary tract, and the retroperitoneum [3,4]. Two groups of RMS are molecularly classified based on the presence or absence of the *PAX3/7-FOXO1* fusion gene: fusion-positive or fusion-negative RMS (FPRMS or FNRMS). The chromosomal translocations t(2;13)(q35;q14) and t(1;13)(p36;q14) are characteristic of FPRMS, resulting in the presence of *PAX3-FOXO1* or *PAX7-FOXO1* fusion proteins [5]. Fusion-negative RMS constitutes a heterogeneous group that often shows point mutations involving the proto-oncogene RAS family proteins [6].

Heparan sulfate proteoglycans (HSPGs) contain one or more covalently attached heparan sulfate (HS) chains, which are a type of glycosaminoglycan. HSPGs are ubiquitously found on the cell surface, such as glypicans and syndecans (SDCs), and in the extracellular matrix (ECM), such as perlecan or agrin. They are essential regulators of cell signaling and tumor progression due to their ability to modulate growth factor interactions, cell adhesion, and migration. Due to the diverse structure of HS chains and their polyanionic characteristics, they can recruit and interact with a wide range of molecules, such as chemokines, ECM components, hormones, growth factors, and enzymes [7,8].

SDCs are transmembrane HSPGs consisting of four members (syndecan-1-4, SDC1-4) in vertebrates [9,10]. The transmembrane localization of SDCs establishes a physical link between the actin cytoskeleton and the ECM, and they also play a role in several “inside-out” and “outside-in” signaling processes. SDC4 is universally expressed in virtually all cell types, and it is highly present on satellite cells [11]. SDC4 interacts with the actin cytoskeleton and cell adhesion proteins, thereby contributing to the regulation of cell adhesion and migration [12]. Earlier, we demonstrated that SDC4 affects myogenesis via small GTPase Rac1-mediated actin cytoskeleton remodeling [13]. Rac1 plays a role in cell proliferation and migration and in tumor development, invasion, and metastasis formation [14]. By affecting several signaling pathways and biological processes such as cell proliferation, migration, metastasis formation, and angiogenesis, SDC4 is also highly involved in tumor progression and development. SDC4 is overexpressed in several tumors, e.g., in melanoma, breast cancer, and osteosarcoma [15]. Previously, we reported that SDC4 is overexpressed in FNRMS, where it may serve as a tumor driver gene [13].

Trastuzumab is a humanized recombinant monoclonal antibody targeting the juxtamembrane domain of the human epidermal growth factor receptor-2 (HER2). Upon receptor binding, the expression of the receptor is reduced [16]. Trastuzumab is mostly used to treat breast cancer with an overexpression of HER2 (HER2-positive breast cancer). Importantly, trastuzumab can decrease the expression of SDC4 in anoikis-resistant endothelial cells [17]. It has been reported that both FPRMS and FNRMS frequently show increased expression of HER2 [18,19,20]. Up to now, no previous study has investigated the effects of trastuzumab treatment in RMS; however, a trastuzumab–deruxtecan (topoisomerase 1 inhibitor) conjugate has been tested in different pediatric malignancies, including RMS [21].

Since FNRMSs express high levels of SDC4, and since SDC4 expression is decreased by trastuzumab in endothelial cells, it is a plausible idea to study the effect of trastuzumab on FNRMS cells. We further analyze the expression of various HSPGs in human RMS samples. Trastuzumab decreases the expression of SDC4 in RD (FNRMS) cells; decreases the expression of cyclin E and cyclin D1, regulators of the G1/S checkpoint; and decreases the cell number. Our results contribute to the understanding of the role of HSPGs in FNRMS.

## 2. Results

### 2.1. Copy-Number Alterations and mRNA Expression Levels of HSPGs in Human RMS Samples

FNRMSs constitute a heterogeneous group in which point mutations have been identified, but only limited information is available about their pathogenesis. We showed earlier that the SDC4 gene was amplified in 28% of human FNRMS samples and in only 14% of FPRMS samples. SDC4 also exhibited a significantly higher mRNA expression level in the fusion-negative group, suggesting that SDC4 is a potential tumor driver gene for FNRMS [13]. Based on these results, we aimed to investigate the copy-number alterations and mRNA expression levels of different HSPGs in human RMS samples.

**Figure 1:**
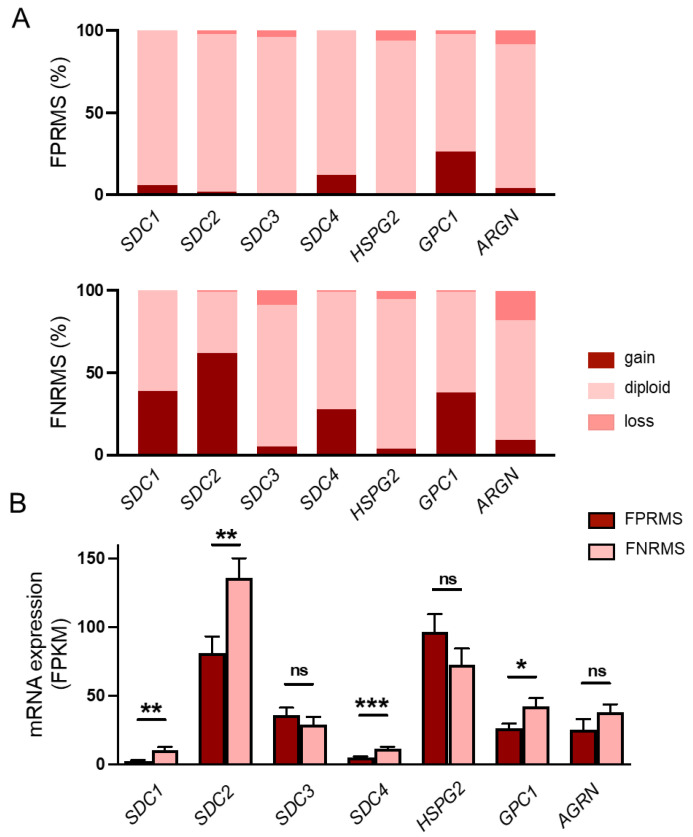
Copy-number changes and mRNA levels of heparan sulfate proteoglycans in human rhabdomyosarcoma (RMS) samples. (**A**) Copy numbers of syndecan-1-4 (*SDC1-4*), perlecan (*HSPG2*), glypican-1 (*GPC1*), and agrin (*AGRN*) genes were analyzed. Gene amplifications were detected in case of *SDC1* (38%), *SDC2* (62%), *SDC4* (28%), and *GPC1* (38%) in FNRMS samples. n = 199 RMS samples; n = 150 fusion-negative RMS (FNRMS) and n = 49 fusion-positive RMS (FPRMS) samples. (**B**) RNA sequencing was performed, and *SDC4* mRNA expression levels of FNRMS (n = 29) and FPRMS (n = 8) samples were quantified; mRNA overexpression of *SDC1*, *SDC2*, *GPC1*, and *SDC4* was detected in the FNRMS group; unpaired *t* test, mean + SEM; ns: non-significant; * *p* < 0.05; ** *p* < 0.01; *** *p* < 0.001.

Genomic analyses of the examined HSPGs revealed copy-number amplification of *SDC1* in 38% (58 out of 150), *SDC2* in 62% (93 out of 150), and glypican-1 (*GPC1*) in 38% (57 out of 150) of the fusion-negative tumors (Figure 1A, Supplementary Table S1). No notable gene amplification was detected for *SDC3* (5%), perlecan (*HSPG2*, 4%), or agrin (*AGRN*, 9%) (Figure 1A, Supplementary Table S1). Based on the mRNA sequencing data, the copy-number amplifications were accompanied by significantly higher *SDC1*, *SDC2*, and *GPC1* mRNA expression in the FNRMS group (Figure 1B), suggesting that these HSPGs, in addition to *SDC4*, may also play a role in the tumorigenesis of FNRMS.

### 2.2. The Phosphorylation of Ser179 of SDC4 Decreases in RD Cells, Accompanied by a High Rac1-GTP Level

Earlier, we demonstrated that SDC4 affects myogenesis via Rac1-mediated actin remodeling [13]. SDC4 regulates the activity of the small GTPase Rac1, and the activation depends on both the expression of SDC4 [13] and the phosphorylation of Ser179 of the cytoplasmic domain of SDC4 [22]. Because of the multiple roles of Rac1 in tumorigenesis and the high expression of SDC4 in FNRMS, next, we wanted to investigate how the activity and the expression of Rac1 change in RD cells compared to myoblasts and myotubes.

**Figure 2:**
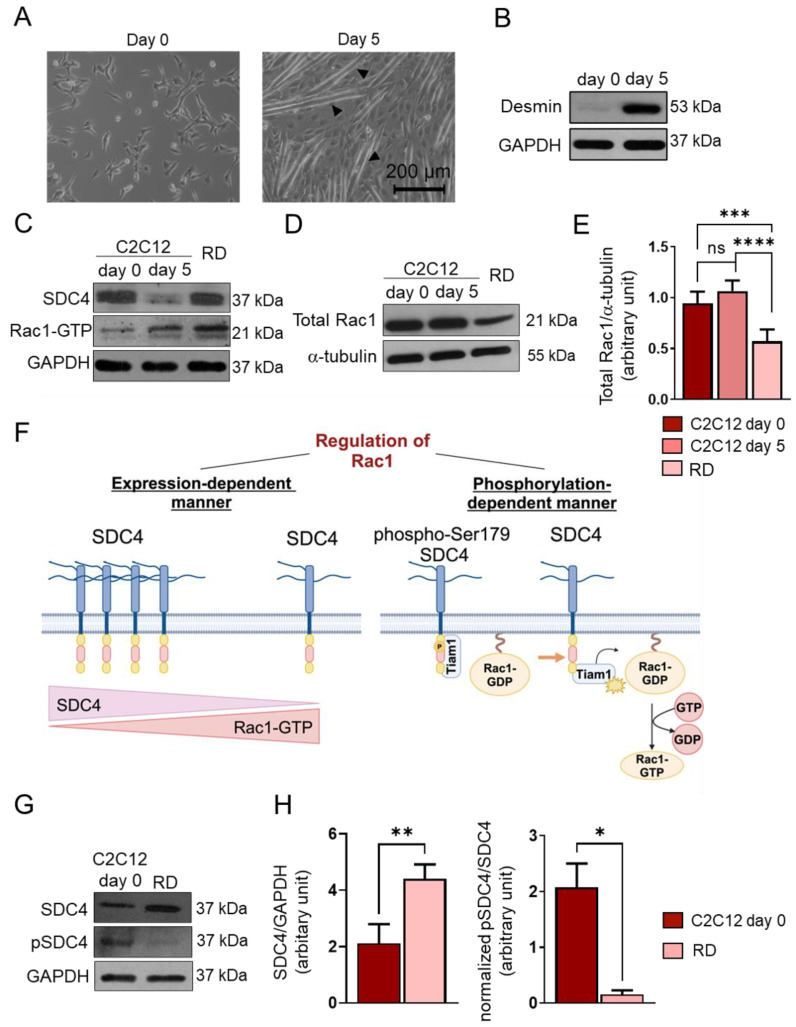
Changes in syndecan-4 (SDC4) phosphorylation and Rac1 GTPase expression and activity. (**A**) Representative phase-contrast images of proliferating (day 0) C2C12 myoblasts and differentiated (day 5) myotubes. Arrowheads show myotube formation. (**B**) A Western blot experiment shows the level of desmin in C2C12 myoblasts and differentiated (day 5) myotubes. GAPDH was used as a loading control. (**C**) Western blot experiments depict the expression of SDC4 in proliferating C2C12 myoblasts (day 0), differentiated myotubes (day 5), and RD (FNRMS, fusion-negative rhabdomyosarcoma) cells. GAPDH was used as a loading control. (**D**) A representative Western blot experiment shows the expression of total Rac1 in C2C12 myoblasts, myotubes, and RD cells; α-tubulin was used as a loading control. (**E**) A quantification of the Western blot results in panel (**D**) is presented; the protein levels were normalized to that of the loading control; n = 6 independent experiments; one-way ANOVA; mean + SEM; ns: non-significant; *** *p* < 0.001; **** *p* < 0.0001. (**F**) A schematic representation of the SDC4-dependent regulation of Rac1 GTPase activity. (**G**) A representative Western blot compares the levels of SDC4 and phospho-Ser179SDC4 in C2C12 myoblasts and RD cells. (**H**) A quantification of the Western blot results in panel (**G**) is presented; n = 3 independent experiments; unpaired *t* test; mean + SEM; * *p* < 0.05; ** *p* < 0.01.

After 5-day-long differentiation of C2C12 myoblasts, myotubes formed (Figure 2A). To monitor the myoblast differentiation, we characterized the amount of desmin expressed by the myoblasts and myotubes (Figure 2B). Desmin was barely detectable in the day 0 sample, and its expression was high at day 5, indicating proper differentiation (Figure 2B).

Next, we monitored the Rac1-GTP levels using a pull-down assay with the p21-binding domain of PAK1 and the levels of SDC4 in proliferating C2C12 cells (day 0), differentiated myotubes (d5), and RD cells (Figure 2C). During muscle differentiation (day 5 vs. day 0), the decreased level of SDC4 allowed for Rac1 activation. However, in RD cells, high SDC4 expression was associated with high Rac1-GTP (Figure 2C).

We also found that the total Rac1 expression appeared to be significantly lower in RD cells compared to C2C12 myoblasts (d0) and myotubes (d5) (Figure 2D,E). Therefore, high Rac1 activity was accompanied by low total Rac1 levels in RD cells.

Since the phosphorylation of Ser179 of SDC4 decreases Rac1 activity by regulating the interaction of the guanine nucleotide exchange factor Tiam1 (T-cell lymphoma invasion and metastasis-inducing protein 1) and Rac1 [22] (Figure 2F), next, we investigated the levels of phosphoSer179 of SDC4 and total SDC4 in C2C12 myoblasts and RD cells (Figure 2G,H). Although the expression of SDC4 was significantly higher in RD cells, only an insignificant amount was actually phosphorylated, meaning that the pSDC4/SDC4 ratio was significantly lower in RD cells compared to myoblasts (Figure 2H). This may explain why we found both high SDC4 expression and Rac1 activation in RD cells.

### 2.3. Trastuzumab Treatment Decreases SDC4 Expression

Since it has been reported that trastuzumab can reduce the expression of SDC4 in anoikis-resistant endothelial cells [17], and we have shown that FPRMS and RD cells express high levels of SDC4, next, we investigated the effects of trastuzumab on RD cells.

**Figure 3:**
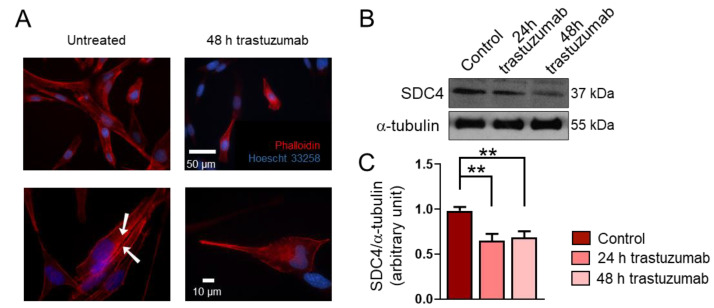
The effect of trastuzumab on the syndecan-4 (SDC4) expression of RD cells. (**A**) Representative fluorescent images show the phenotype of untreated and trastuzumab-treated (20 μg/mL, 48 h) RD cells. Red: Alexa-647-conjugated phalloidin, blue: Hoechst 33258. Arrowheads show actin stress fiber formation. (**B**) A representative Western blot experiment shows the levels of SDC4 in control cells and following 24 h and 48 h of trastuzumab (20 μg/mL) treatment. α-tubulin was used as a loading control. (**C**) A quantification of the results of panel (**B**) is shown; the protein levels were normalized to that of α-tubulin; n = 3 independent experiments; one-way ANOVA; mean + SEM; ** *p* < 0.01.

Data from several studies suggest that RMSs express high levels of HER2 [18,19,20]; however, HER2’s role in the genesis of RMS is unknown. Importantly, RD cells express HER2 [23]. During a 48 h treatment period, trastuzumab administration resulted in morphological changes in RD cells (Figure 3A). Untreated cells showed stress fiber formation characteristic of migrating cells (Figure 3A). Notably, the trastuzumab treatment decreased the expression of SDC4 in RD cells (Figure 3B,C).

### 2.4. Trastuzumab Administration Reduces Cyclin E and Cyclin D1 Levels and Decreases Cell Number

**Figure 4:**
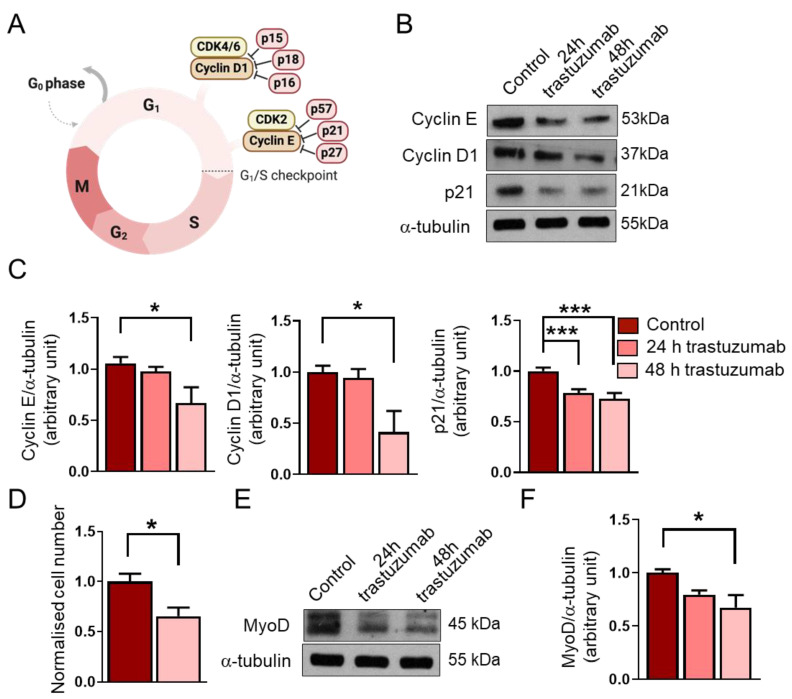
The effects of trastuzumab treatment on RD cells. (**A**) A schematic representation of the G1/S checkpoint and its regulators. (**B**) A representative Western blot experiment shows the levels of cyclin E, cyclin D1, and p21 in untreated RD cells and following 24 h and 48 h trastuzumab (20 μg/mL) treatments. α-tubulin was used as a loading control. (**C**) A quantification of the results in panel (**B**) is shown; equal amounts of proteins were loaded, and the protein levels were normalized to that of α-tubulin; n = 4 independent experiments, one-way ANOVA; mean + SEM; * *p* < 0.05; *** *p* < 0.001. (**D**) After a 48 h trastuzumab treatment, both treated and untreated cells were counted; cell numbers were normalized to the control cell number; n = 6 independent experiments, unpaired *t* test; mean + SEM; * *p* < 0.05. (**E**) A representative Western blot experiment shows the levels of MyoD in untreated RD cells and following trastuzumab treatment. α-tubulin was used as a loading control. (**F**) A quantification of the results in panel (**E**) is shown; the protein levels were normalized to that of α-tubulin; n = 4 independent experiments; one-way ANOVA; mean + SEM; * *p* < 0.05.

High expression of SDC4 can increase the proliferation rate of cells due to its co-receptor role for growth factors. Moreover, earlier, we showed that decreased SDC4 expression in C2C12 myoblasts results in decreased proliferation of these cells [24]. Consequently, next, we investigated how trastuzumab treatment could affect the expression of cell cycle markers (Figure 4A–C).

We found that a 48 h trastuzumab treatment decreased the expression of the G1/S checkpoint regulators cyclin E and cyclin D1 (Figure 4B,C). In accordance with these results, we also observed a significant reduction in cell number after trastuzumab treatment for 48 h (Figure 4D).

In addition, we aimed to investigate the level of the cyclin-dependent kinase inhibitor p21. Despite the decreased levels of cyclin E and cyclin D1 and the reduced cell number, we found that the expression of p21 decreased after the trastuzumab treatment (Figure 4B,C). Interestingly, this reduction in the p21 level was observed after 24 h, whilst decreased cyclin E and cyclin D1 levels could be detected after 48 h. The longer trastuzumab treatment (48 h) did not result in a more significant decrease in the p21 level.

Several recent studies have revealed that the myogenic transcription factor MyoD is highly expressed in RMS, and although it cannot support differentiation, as it does during myogenesis, it is essential for the survival and proliferation of tumor cells [25]. Therefore, an objective of this study was also to investigate how trastuzumab would affect the expression of MyoD in RD cells. We found that the 48 h trastuzumab treatment reduced the expression of MyoD in RD cells (Figure 4E,F).

## 3. Discussion

RMS is the most common soft tissue sarcoma in children; as of today, despite advances in both diagnosis and therapy improving the survival rates of RMS, the overall 3-year survival rate for the metastatic form is still less than 30% [2]. The treatment for RMS involves a combination of multi-agent systemic chemotherapy to eliminate widespread disease and surgical removal of the primary tumor, which can be supplemented with radiotherapy to ensure effective local disease control. Due to the low survival rate of the metastatic form despite the use of high doses of chemotherapy, several agents are being investigated to improve the outcome; examples include cixutumab (monoclonal antibody targeting the type 1 human insulin-like growth factor-1 receptor) and pazopanib (Tyr-kinase inhibitor) [26,27].

HSPGs play a role in interacting with growth factors, chemokines, and structural proteins of the ECM to influence proliferation, differentiation, and other important cellular functions. HSPGs are also highly investigated in cancer research due to their diverse roles in cancer progression and metastasis formation. Despite this growing body of research, the role of HSPGs in RMS is still not well understood. HSPGs act as co-receptors for growth factors, thereby amplifying oncogenic signaling pathways that contribute to cell proliferation, invasion, and angiogenesis in RMS as well [28].

Interestingly, the expression of SDC1 and SDC2 is not characteristic of skeletal muscle; however, in FNRMS samples, gene amplifications and mRNA overexpression of SDC1 and SDC2 were detected. SDC1 is mostly expressed in epithelia and plasma cells, whilst SDC2 is widely present in mesenchymal cells, such as fibroblasts and smooth muscle [9]. In contrast, SDC4 is mainly expressed in myoblasts, and its expression decreases during myogenesis; myotubes express only low levels of SDC4. However, SDC4 gene amplification and mRNA overexpression were observed in FNRMS samples. SDC4 is known to play a role in cell adhesion, proliferation, and migration, which makes it essential for cancer development, invasion, and metastasis in several cancer types [10]. Among the SDC family members, SDC3 is characteristic of mature skeletal muscle, but neither FPRMS nor FNRMS samples showed gene amplification.

SDC1 was amplified in 38% of the human FNRMS samples, compared to only 6% of FPRMS samples. This amplification was coupled with significantly higher SDC1 mRNA expression in the fusion-negative group. Zeng et al. reported that SDC1 helps to regulate the MMP-7/syndecan-1/TGF-β1 autocrine loop in hepatocellular carcinoma [29]. The TGF-β autocrine loop has been previously reported to be involved in RMS [30], so it is likely that such links exist between SDC1 and RMS. SDC1 is overexpressed in several cancer types, such as prostate cancer, colorectal cancer, and glioblastoma [31]. In our study, SDC2 was amplified in 62% of the FNRMS samples, with overexpression of SDC2 mRNA, compared to 2% of fusion-positive samples. SDC2 has been reported to play a role in Notch signaling. Studies show that Notch receptors induce the expression of SDC2, which enhances Notch signaling in vascular smooth muscle differentiation [32]. Notch signaling has also been reported by Conti et al. to play a crucial role in the development and progression of RMS [33], which could explain our findings. Syndecan-2 is associated with tumor progression and invasiveness in several types of cancers, such as melanoma, fibrosarcoma, and breast cancer [34].

We also found that GPC1 was amplified in 38% of the fusion-negative group and was associated with significantly higher glypican-1 mRNA expression, while only 26% of the FPRMS samples showed gene amplification. Although direct studies on the role of GPC1 in RMS are scarce, in other cancers, such as breast cancer or pancreatic cancer, GPC1 is involved in the regulation of several signaling pathways like FGF or TGF-β signaling. Dysregulation of these pathways could lead to impaired cell proliferation, differentiation, or migration [35].

**Figure 5:**
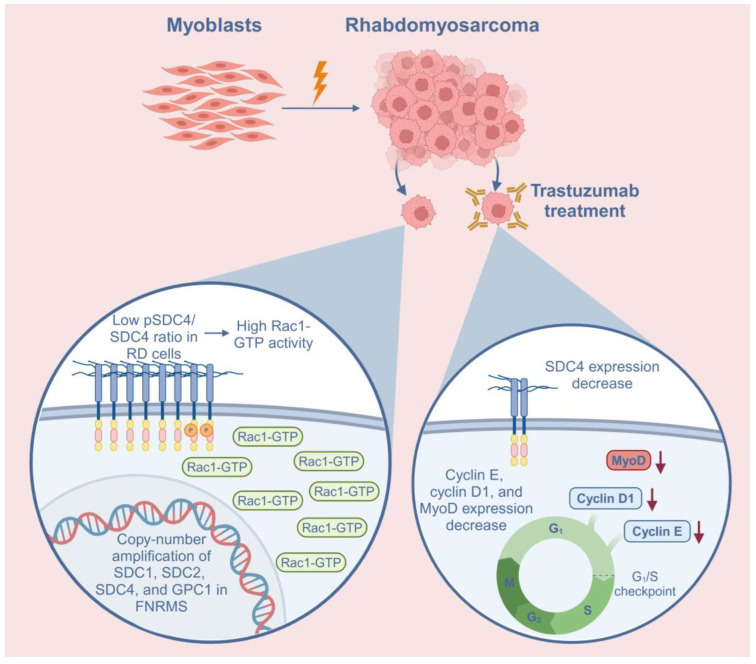
Schematic summary of the role of syndecan-4 (SDC4) in rhabdomyosarcoma (RMS) and the effects of trastuzumab treatment on RD cells. In addition to SDC4, syndecan-1 (SDC1), syndecan-2 (SDC2), and glypican-1 (GPC1) heparan sulfate proteoglycans demonstrated copy-number amplifications in fusion-negative RMS (FNRMS), indicating that they may have a role in tumorigenesis. The low phosphoSer179-SDC4/SDC4 (pSDC4/SDC4) ratio causes high Rac1-GTP activation in RD cells. Trastuzumab, a humanized monoclonal antibody targeting HER2, decreased the expression of SDC4 in RD cells. Trastuzumab treatment also decreased the expression of cyclin E, cyclin D1, and MyoD, suggesting cell cycle arrest. Red arrows indicate the decreased levels of the proteins.

Rac1 is a member of the Rho family of small GTPases, which are known to alternate between an active GTP-bound form and an inactive GDP-bound form, controlled by guanine nucleotide exchange factors (GEFs), GTPase-activating proteins (GAPs), and guanine nucleotide dissociation inhibitors (GDIs) [16]. Rac1 is known to be the key regulator of the actin cytoskeleton and participates in signaling pathways such as MAPK or PI3K/Akt, promoting cell proliferation [36]. Rac1 is often overexpressed or over-activated in several cancers, promoting cell invasion, metastasis, and angiogenesis [37]; however, the expression of Rac1 in RMS was previously unknown. Since our prior study showed that during muscle differentiation, the activation of Rac1 also depends on the expression of SDC4, we aimed to investigate the levels of SDC4, Rac1-GTP, and total Rac1 in RD cells. We found that Rac1-GTP levels were high in RD cells, potentially promoting tumor cell proliferation and migration. Since the phosphoSer179-SDC4/SDC4 ratio was significantly lower in RD cells compared to C2C12 myoblasts, low levels of phosphoSer179 of SDC4 contributed to high Rac1 activity in RD cells (Figure 5). The high Rac1-GTP level may have resulted in low total Rac1 levels in the RD cells through negative feedback.

HER2 is a transmembrane receptor of the ErbB family of tyrosine kinase receptors, playing a critical role in cell growth, differentiation, and survival. It exerts its effects through key oncogenic pathways, including PI3K/AKT and RAS-MAPK, which also regulate proliferation and tumor progression [38]. While HER2 is widely studied in HER2-positive breast cancer, it is also overexpressed in several other malignancies, such as ovarian, gastric, and bladder cancers [39,40,41]. HER2 is also reported to be overexpressed in RMS, suggesting a potential role in tumorigenesis [19,20,23]. Notably, experimental models demonstrate that the activation of the HER2 oncogene coupled with the inactivation of the oncosuppressor gene p53 causes RMS in mice [18].

It has been reported that trastuzumab, a monoclonal recombinant antibody targeting HER2 that is commonly used to treat HER2-positive breast cancer, reduces the expression of SDC4 in anoikis-resistant endothelial cells [17]. In line with these findings, we observed that trastuzumab reduced the expression of SDC4 in RD cells. Moreover, the RD cells exhibited high Rac1-GTP levels, an elongated phenotype, and actin stress fiber formation; stress fiber formation was not observed after the trastuzumab treatment, indicating that the migration of these cells may decrease after trastuzumab administration.

Trastuzumab decreased the expression of the G1/S checkpoint regulators cyclin E and cyclin D1 in RD cells; however, the expression of cyclin-dependent kinase inhibitor p21 was also reduced after trastuzumab treatment (Figure 5). This rather contradictory result may be due to the degradation of p21, since it has been reported that in RMS, MyoD, the major myogenic transcription factor, binds ubiquitin ligase SKP2, which ubiquitylates cyclin-dependent kinase inhibitors such as p21, p27, and p57, thereby targeting them for degradation in RMS [42]. Trastuzumab has also been reported to downregulate p21 in TC-71 cells (Ewing’s sarcoma), potentially through decreasing MAP/ERK kinase activation [43]. The effects of trastuzumab treatment on p21 expression appear to vary in different cancer types. For example, in HER2-positive breast cancer, the loss of p21 expression is mediated through HER2/HER3 heterodimerization and associated with poor prognosis in patients treated with adjuvant trastuzumab [44]. At present, there is no information on HER2/SDC4 signaling or the effects of trastuzumab on RMS. Importantly, despite low p21 levels, the cell number decreased after trastuzumab treatment, in accordance with the low cyclin E and cyclin D1 levels.

The cell number decreased after the trastuzumab treatment, suggesting that decreased cyclin E and cyclin D1 levels may, at least in part, lead to decreased proliferation; however, we cannot exclude an apoptosis-mediated decrease in cell number. The observed decrease in cell number parallels our earlier observations that decreased SDC4 expression in C2C12 myoblasts results in a decreased proliferation rate [24].

RMS arises from skeletal muscle cells that fail to differentiate terminally. The majority of RMSs express the transcription factor MyoD, which is essential for skeletal muscle differentiation. In RMS, highly expressed MyoD is essential for the survival and proliferation of the tumor cells [25]. Importantly, trastuzumab treatment reduced the expression of MyoD in RD cells. Currently, there has been no study explaining the mechanism of MyoD suppression due to trastuzumab treatment. Whether or not MyoD suppression is beneficial needs to be investigated further.

While this study provides a deeper insight into the molecular background of FNRMS and the role of SDC4 and other HSPGs in it, a few limitations should be considered. Firstly, the genetic analysis of SDC4 and other HSPGs was conducted on a large patient cohort; however, we did not study the correlations between gene amplification and mRNA expression. Furthermore, although trastuzumab treatment was shown to reduce SDC4 expression, the exact molecular mechanism by which trastuzumab regulates the SDC4 level remains unclear. Additionally, the effectiveness of trastuzumab at the applied dose also remains uncertain, since it has not been tested on RMS before, suggesting that dose optimization studies are needed. Addressing these limitations in future studies will be crucial to further explore the therapeutic potential of trastuzumab in RMS.

To summarize, our findings contribute to the understanding of the role of SDC4 and other HSPGs in FNRMS. The gene amplification and higher mRNA expression of SDC1, SDC2, and GPC1 in addition to SDC4 may indicate that these HSPGs can also contribute to the pathogenesis of FNRMS. The trastuzumab-mediated decrease in the expression of SDC4, cyclin E, and cyclin D1 and the trastuzumab-mediated decrease in cell number could have potential importance in RMS treatment in the future.

## 4. Materials and Methods

### 4.1. Copy-Number Analysis and RNA Sequencing of Human Rhabdomyosarcoma Samples

Genomic data were obtained from 199 specimens, each corresponding to a unique patient. These samples were deidentified prior to use and sourced from three datasets: the National Cancer Institute, the Children’s Oncology Group, and the University of Texas Southwestern (UTSW). The genomic analyses were carried out at UTSW Medical Center following approval by its institutional review board (STU 102011-034). The raw genomic data have been submitted to the dbGAP database under accession number phs000720. Whole-genome and whole-exome sequencing reads were aligned to the human reference genome (hg19), and somatic mutations affecting protein function were detected using the Genome Analysis Tool Kit pipeline. SNP arrays underwent processing with the SNP-FASST segmentation algorithm in Nexus BioDiscovery software version 7 (BioDiscovery, El Segundo, CA, USA). Significant copy-number variations (CNVs) were identified using the GISTIC method, applying a q-value cutoff of 0.25 for statistical significance. For gene expression profiling, RNA was analyzed using the Affymetrix Exon 1.0 ST array system in accordance with the manufacturer’s protocols (Thermo Fisher Scientific, Waltham, MA, USA). CEL file data were processed using open source R/BioConductor software version 3.2 (https://www.bioconductor.org/), incorporating robust multiarray average normalization and custom PERL scripts.

### 4.2. Cell Culturing

C2C12 mouse myoblasts (ATCC; Massanas, VA, USA) were maintained in a medium containing 80% Dulbecco’s Modified Eagle Medium (DMEM, 4.5 g/L glucose, L-glutamine, and pyruvate; Lonza, Basel, Switzerland), 20% fetal bovine serum (FBS; Thermo Fisher Scientific), and 65 mg/mL gentamicin. For differentiation, C2C12 cells were cultured in growth medium. After the cells reached 100% confluency, differentiation was induced with differentiation medium containing 98% DMEM and 2% horse serum (Thermo Fisher Scientific) for 5 days.

RD (CCl-136; ATCC) human RMS cells were cultured in 90% DMEM (Lonza)containing 4.5 g/L glucose, L-glutamine, and pyruvate, 10% FBS (Gibco/Thermo Fisher Scientific), and 65 mg/mL gentamicin. All cell lines were maintained in a humified incubator at 37 °C and 5% CO_2_. The passage number of the cells was no greater than 10.

### 4.3. Phase-Contrast Microscopy

The morphology of undifferentiated and differentiated C2C12 cells was documented using representative images captured with a Leica DMi1 phase-contrast microscope (Leica Microsystems, Wetzlar, Germany), equipped with a ×10 objective (Leica Hi Plan ×10, NA = 0.28).

### 4.4. Fluorescence Staining

In order to visualize actin filaments in RD cells, samples were fixed with 4% paraformaldehyde and incubated for 35 min in PBS containing 0.9% Triton X-100 and 4% BSA. The cells were then labeled with Alexa-647-conjugated phalloidin (A22287; Invitrogen, Carlsbad, CA, USA). After the nuclei were stained with Hoechst 33258 (Sigma-Aldrich, St. Louis, MO, USA), the samples were mounted using ProLong Gold Antifade Reagent (9071S; Cell Signaling Technology, Danvers, MA, USA).

Fluorescence images with high resolution were obtained using a Nikon Eclipse Ni-U fluorescence microscope (Nikon Instruments Inc., Melville, NY, USA), equipped with a 100× objective lens (Nikon Plan Apo 100×/1.45 oil, DIC N2).

### 4.5. Trastuzumab Treatment of the Cells

RD cells (170,000 cells/well) were plated on 60 × 10 mm cell culture dishes in growth medium for 24 h. Afterward, the medium was changed, and the cells were treated with 20 µg/mL trastuzumab (Viatris, Canonsburg, PA, USA) according to Onyeisi et al. [17] for 24 or 48 h. The control group was lysed after 48 h. The confluency of the cells before lysis was no greater than 85%.

### 4.6. Assessment of Rac1 GTPase Activity

To measure the activity of Rac1, the cells were lysed in magnesium lysis buffer (25 mM HEPES, pH 7.5, 150 mM NaCl, 1% Igepal CA-630, 10 mM MgCl_2_, 1 mM EDTA, and 2% glycerol) supplemented with 1 mM NaF (Sigma-Aldrich), 1 mM Na_3_VO_4_ (Sigma-Aldrich), and protease inhibitor cocktail (Sigma-Aldrich). The samples were centrifuged at 14,000 rpm for 5 min at 4 °C to eliminate cellular debris. To detect the active Rac1-GTP, the Rac1 Activation Assay Kit (Merck, Darmstadt, Germany) was used, according to the manufacturer’s instructions. In the Rac/cdc42 Assay Reagent, fused to the beads, is the p21-binding domain (PBD) of p21-activated kinase (PAK1), a downstream protein of Rac1, which binds the activated Rac1-GTP. A quantity of 10 mg of the Rac/cdc42 Assay Reagent was added to the samples per 0.5 mL of cell lysates. The samples were incubated for 1 h at 4 °C, during which they were gently stirred. After the incubation, the beads were collected by centrifugation and washed in MLB. Finally, they were resuspended in Laemmli sample buffer and boiled for 5 min. Then, the samples were applied to polyacrylamide gel and transferred onto Protran nitrocellulose membrane (GE Healthcare Amersham, Little Chalfont, Buckinghamshire, UK). The membrane was first incubated with anti-Rac1 antibody (clone 23A8, 05-389; Merck), after which the proper HRP-conjugated secondary antibody was used.

### 4.7. Western Blotting

Cells were lysed in RIPA buffer [20 mM Tris–HCl (pH 7.5), 150 mM NaCl, 1 mM Na2EDTA, 1 mM EGTA, 1% NP-40, 1% sodium deoxycholate, 2.5 mM sodium pyro-phosphate, 1 mM b-glycerophosphate, 1 mM Na3VO4, 1 μg/mL leupeptin; Cell Signaling Technology, #9806] supplemented with 1 mM NaF (Sigma-Aldrich) and protease inhibitor cocktail (Sigma-Aldrich). The cell lysates were centrifuged at 13,000 rpm for 5 min at 4 °C to eliminate cellular debris. The protein concentration in the samples was determined using a BCA protein assay kit (Pierce Chemical, Rockford, IL, USA). A quantity of 30 μg/mL of proteins was loaded per lane in triplicate on a polyacrylamide gel from independent biological samples. Different gel concentrations were used based on the size of the proteins: 12% acrylamide gel was used for p21 and Rac1, and 10% acrylamide gel was used for the rest of the proteins. Gel electrophoresis was performed at 20 mA, followed by transfer onto a Protran membrane (GE Healthcare Amersham) at 35 V for 90 min on ice. Then, the membranes were incubated with the following antibodies: rabbit polyclonal anti-desmin (M076029-2; DAKO, Santa Clara, CA, USA), anti-cyclin E (sc-481; Santa Cruz, Dallas, TX, USA), anti-cyclin D1 (sc-6281; Santa Cruz), anti-p21 (sc-6246; Santa Cruz), anti-MyoD (c-20; sc-377460; Santa Cruz), anti-Rac1 (clone 23A8, 05-389; Merck), anti-phospho-Ser179-SDC4 (PA5-64516; Thermo Fisher Scientific, Waltham, MA, USA), and anti-SDC4 (PA1-32485; Thermo Fisher Scientific). For the control, mouse monoclonal anti-GAPDH (#2118; Cell Signaling Technology, Danvers, MA, USA) and anti-α-tubulin (#T9026; Sigma-Aldrich) were used. After incubation with the appropriate horseradish-peroxidase-conjugated anti-IgG secondary antibodies [anti-mouse (P0161) and anti-rabbit (P0448)] from DAKO, the peroxidase activity was visualized using the enhanced chemiluminescence procedure (Advansta, Menlo Park, CA, USA). Supplementary Table S2 contains the appropriate dilutions of the primary and secondary antibodies that were used. Signal intensities were quantified using the QuantityOne software version 4.6.6 (Bio-Rad, Hercules, CA, USA). Western blot experiments were repeated in triplicate with independent biological samples.

### 4.8. Calculation of Cell Numbers

To determine the effect of trastuzumab treatment on cell numbers, RD cells were treated with 20 µg/mL trastuzumab for 48 h. After Trypan blue staining (Corning, Glendale, AZ, USA) to determine the viable cells in the cell suspension, the cell numbers were counted in Bürker-Türk counting chambers (BRND719505, BLAUBRAND, Wertheim, Germany) and normalized to the control cell number.

### 4.9. Statistical Analysis

Statistical analyses were conducted using GraphPad Prism software version 7.05 (GraphPad Software Inc., San Diego, CA, USA). The unpaired *t* test and one-way ANOVA were applied, followed by pair-wise comparisons using Dunnett’s multiple comparisons test. The data are presented as the mean ± SEM. *p*-values less than 0.05 were considered statistically significant. The individual *p*-values and their corresponding significance levels are displayed on the graphs.

## Data Availability

The data presented in this study are available on request from the corresponding author. The original genomic data were deposited into the dbGAP database with accession number phs000720.

## Supplementary Materials

The following supporting information can be downloaded at: https://www.mdpi.com/article/10.3390/ijms26052137/s1.
