# Supplementary material for: Trastuzumab Decreases the Expression of G1/S Regulators and Syndecan-4 Proteoglycan in Human Rhabdomyosarcoma
Source: Int J Mol Sci. 2025 Feb 27;26(5):2137. doi: 10.3390/ijms26052137 (PMC11900631; doi:10.3390/ijms26052137)
Supplement: Supplementary file 1 [file ijms-26-02137-s001.zip › ijms-3479349-supplementary.pdf]

Table S1: Copy-number data of heparan sulfate proteoglycans of fusion-positive and fusion-negative human rhabdomyosarcoma samples, $n = 199$.

FPRMS	SDC1	SDC2	SDC3	SDC4	GPC1	HSPG2	AGRN
Gain	3	1	0	6	13	0	2
Diploid	46	47	47	43	35	46	43
Loss	0	1	2	0	1	3	4

FNRMS	SDC1	SDC2	SDC3	SDC4	GPC1	HSPG2	AGRN
Gain	58	93	8	42	57	6	14
Diploid	92	56	129	107	92	136	109
Loss	0	1	13	1	1	8	27

Table S2: Applied dilutions of primary and secondary antibodies.

Primary antibody					Secondary antibody			
Name	Carrier animal	Dilution	Cataloge number	Manufacturer	HRP-conjugate	Dilution	Cataloge number	Manufacturer
α-tubulin	rabbit	1:1000	#T9026	Sigma	anti-rabbit	1:1000	P 0448	DAKO
GAPDH	mouse	1:1000	#2118	Cell Signaling Technology	anti-mouse	1:1000	P 0260	DAKO
Desmin	rabbit	1:1000	M076029-2	DAKO	anti-rabbit	1:1000	P 0448	DAKO
SDC4	rabbit	1:1000	PA1-32485	Thermo Fisher Scientific	anti-rabbit	1:1000	P 044	DAKO
Rac1	mouse	1:1000	clone 23A8, 05-389	Merck	anti-mouse	1:2500	P 0260	DAKO
Phospho-Ser179-SDC4	rabbit	1:500	PA5-64516	Thermo Fisher Scientific	anti-rabbit	1:2000	P 044	DAKO
Cyclin E	rabbit	1:500	sc-481	Santa Cruz	anti-rabbit	1:1000	P 044	DAKO
Cyclin D1	mouse	1:300	sc-6281	Santa Cruz	anti-mouse	1:1000	P 0260	DAKO
p21	mouse	1:500	sc-6246	Santa Cruz	anti-mouse	1:1000	P 0260	DAKO
MyoD	rabbit	1:500	sc-377460	Santa Cruz	anti-rabbit	1:1000	P 044	DAKO
